# High smoking and low cessation rates among patients in treatment for opioid and other substance use disorders

**DOI:** 10.1186/s12888-022-04283-6

**Published:** 2022-10-19

**Authors:** Endre Dahlen Bjørnestad, John-Kåre Vederhus, Thomas Clausen

**Affiliations:** 1 Addiction Unit, Sørlandet Hospital HF, Po. box 416, N-4604 Kristiansand, Norway; 2 Norwegian Centre for Addiction Research (SERAF), University of Oslo, Kirkeveien 166, N-0407 Oslo, Norway; 3 Addiction Unit, Sørlandet Hospital HF, Po. box 416, N-4604 Kristiansand, Norway

**Keywords:** Opioid maintenance treatment, SUD Treatment, Tobacco, Smoking cessation

## Abstract

**Background:**

Smoking is a well-documented cause of health problems among individuals with substance use disorders. For patients in opioid maintenance treatment (OMT), the risk for somatic health problems, including preventable diseases associated with tobacco smoking, increases with age. Our aim was to describe smoking among patients entering substance use disorder (SUD) treatment, investigate changes in smoking from the start of treatment to 1-year follow-up, and explore factors related to smoking cessation.

**Methods:**

We employed data from the Norwegian Cohort of Patient in Opioid Maintenance Treatment and Other Drug Treatment Study (NorComt). Participants were 335 patients entering SUD treatment at 21 participating facilities across Norway. They were interviewed at the start of treatment and at 1-year follow-up. The main outcomes were smoking and smoking cessation by treatment modality. A logistic regression identified factors associated with smoking cessation.

**Results:**

High levels of smoking were reported at the start of treatment in both OMT (94%) and other SUD inpatient treatment patients (93%). At 1-year follow-up most patients in OMT were still smoking (87%), and the majority of the inpatients were still smoking (69%). Treatment as an inpatient was positively associated and higher age was negatively associated with smoking cessation. Most patients who quit smoking transitioned to smokeless tobacco or kept their existing smokeless habit.

**Conclusion:**

As illustrated by the high smoking prevalence and relatively low cessation levels in our sample, an increased focus on smoking cessation for patients currently in OMT and other SUD treatment is warranted. Harm-reduction oriented smoking interventions may be relevant.

**Supplementary Information:**

The online version contains supplementary material available at 10.1186/s12888-022-04283-6.

## Background

The burden of disease associated with opioid use disorder (OUD) is substantial [1, 2]. Mortality among patients in opioid maintenance treatment (OMT) is related not only to overdose deaths [3] but also to somatic and infectious diseases that are treatable if detected and handled appropriately [4, 5]. Smoking is a well-documented cause of health problems for individuals with substance use disorders (SUDs) in general [6–8]. Individuals with opioid use disorders appear especially at risk, given established associations between opioid and nicotine use [9–11]. Research has indicated that smoking has generally been higher among individuals with substance use disorder compared with the general population [12]. Whilst prevalence of cigarette smoking in the general population has declined over the past decades [13], smoking rates appear to have remained relatively high among individuals with SUDs including OUD, both inside and outside treatment [8, 14]. Smoking cessation intervention is generally recommended for individuals with SUD [15], but also specifically for patients with OUD [7, 16]. Research has suggested that patients with OUD in treatment are motivated to cease smoking when asked [7]. In clinical practice several treatment options are, in principle, available [17]. At the same time, individuals with OUD and in OMT who are attempting to quit smoking face extra challenges, such as nicotine opioid interactions [9], complex needs [18], and a range of other individual-level challenges that may demand special attention from treatment providers [7].

Some countries have ageing OMT populations [19, 20], and this can be considered an indicator of the success of OMT [21, 22]. However, the burden of somatic health problems among individuals with OUD, including those receiving OMT, increases with age [23–25]. Older patients in OMT have reported a higher prevalence of somatic comorbidities [26, 27] and sub-clinical, self-reported impairments [28, 29]. Lifestyle-related factors such as smoking [3] that contribute to somatic diseases among ageing OMT patients [25] are therefore of concern.

As OMT populations age, the need for more descriptive information on smoking is important in the design of appropriate preventive programs.

The aim of the present study was to explore smoking rates and smoking cessation following SUD-treatment entry among patients in OMT and other SUD treatment. Specifically, we aimed to.

1. Describe smoking among patients entering SUD treatment (baseline).
2. Investigate change in smoking between start of treatment (T0) and 1-year follow-up (T1) by treatment modality, contrasting patients that entered OMT with patients that entered inpatient treatment.
3. Explore factors associated with smoking cessation in OMT and other SUD treatment.

## Methods

### Study design

Data were drawn from the Norwegian Cohort of Patients in Opioid Maintenance Treatment and Other Drug Treatment (NorComt) study [30]. NorComt is a longitudinal, naturalistic, multi-site study that was designed to increase understanding of factors influencing treatment adherence and outcomes for a diverse patient population across standard care treatment modalities (OMT and other inpatient SUD treatments). For this study, our primary patient group of interest comprised individuals who entered OMT. Inpatients served as a comparison treatment group, to put the results from OMT into context.

### Setting

There were 21 participating facilities across Norway: 14 were OMT outpatient centers, and 7 were inpatient centers (predominately non-OMT). OMT in Norway is generally provided on an outpatient basis by publicly funded health services, following a national treatment guideline [31]. The 2010–2022 OMT guideline was in use when the data for the present study were collected. Although the specialist healthcare service serves as the overall responsible provider, the treatment is provided in collaboration with primary healthcare and social services. Apart from an established opioid use disorder diagnosis, there are no further criteria for entering this type of treatment, although substitution-free treatment is generally recommended as a first option. Inpatient SUD treatment represented in the present project typically has a duration of 6–9 months, primarily with non-OMT treatment in therapeutic community-like settings. Some patients transfer to an outpatient treatment after completing inpatient treatment. There are no specific regulations on tobacco use in Norwegian SUD treatment, other than compliance with Norwegian legislation restricting smoking inside public buildings, including health institutions. This means that, when receiving either type of treatment, patients would need to smoke outdoors, for example in a designated area in close proximity to the treatment site. The study setting has been described in more detail in previous publications [32, 33].

### Participants

To participate, the only formal inclusion criterion was admittance to a SUD treatment facility, and there were no formal exclusion criteria. Participants were consecutively enrolled in the study when beginning treatment (N = 548) and consented at baseline to be contacted for additional data collection one year later for a follow-up interview [30]. Clinicians at each respective treatment center conducted the baseline interviews of consecutively enrolled patients within an average of 3 weeks from treatment initiation. The interview questions were framed to reflect the period prior to the start of treatment. Thus, “T0” refers to the start of treatment for each specific patient. There were 341 participants included at follow-up (62% of initial participants). Of these, 335 (61%) had measurements of smoking at both T0 and T1 and were included in the further analysis.

### Measures

A structured interview included questions on sociodemographic variables, substance use, and a variety of measurements ranging from mental health to quality of life [32, 33]. The main outcomes were smoking and whether patients reported smoking cessation. Participants were asked both at T0 and T1 whether they had smoked cigarettes the past 6 months or not. At both assessments, they were also asked to estimate the number of cigarettes smoked per day. It was therefore possible to explore whether smoking status had changed from baseline to follow-up. Patients were also asked whether they used smokeless tobacco or not, as well as how many days one box lasted: a low number of days per box implies higher use intensity. Excerpts from the EuropASI, a validated version of the Addiction Severity Index adapted for European use [34], were used to collect data on most used substances or addictive medications. Only data on the four most used substance types were collected. The Severity of Dependence Scale (SDS) [35] was employed as a measure of psychological dependence, and as a severity measure. The SDS is a validated five-item scale that was designed to measure dependence on specific substances (e.g., “Did you think your use of heroin was out of control?”), but here the items have been rephrased to reflect general dependence on substances (e.g., “Did you think your use of substances was out of control?”). Responses were given in a 4-point format ranging from 0 to 3, with 0 corresponding to “Never” and 3 corresponding to “Always”. The summed scale ranged from 0 to 15, with higher scores representing higher severity. The Hopkins Symptom Checklist 25-item version (HSCL-25) [36] was included as a measure of mental distress [37]. The version employed in our study used a 5-point Likert-type response format [38, 39]. Each participant’s mean score (range 0–4) was used in the analysis. A score of 1.0 indicates mental distress of clinical concern [39, 40]. Somatic health-related variables were selected for analysis primarily based on their relevance to smoking. Self-reported physical health was measured by an item from the QOL10 [41], and patients were asked how they considered their physical health prior to treatment in a 5-point Likert-type format ranging from 0 to 4, with 0 corresponding to “very poor” and 4 to “very good”. Somatic health complaints were self-reported using a structured questionnaire that included multiple complaints. Patients were asked to indicate the degree which they “had been bothered by respiratory ailments in the past 2 weeks” on a 5-point scale from 0 to 4, with 0 corresponding to “not at all”, 1 “a little”, 2 “moderately”, 3 “a lot”, and 4 “very much”.

### Analysis strategy/Statistical analysis

Participant sociodemographic data, substance-use variables and health-related variables were summarized using descriptive statistics. Continuous variables are reported as means (M) and standard deviations (SD), or medians (Mdn) and interquartile ranges (IQR). Categorical variables are reported as frequencies and percentages. As we were interested in tobacco smoking by treatment modality, we tested differences in background variables at baseline between patients in OMT and inpatient treatment with t-tests or chi-square tests, depending on variable type (continuous or categorical). Categorical outcomes within the full sample and within each treatment modality were tested using McNemar’s test. Changes in continuous outcomes were tested using the matched-pairs Wilcoxon signed-rank test, as the data deviated from normal distribution. Associations between relevant independent factors and smoking cessation were investigated with bivariate logistic regression. We calculated the differences between follow-up and baseline and recoded the resulting data into a dichotomous variable where 0 was “no change” or “started smoking”, and 1 represented a change in smoking, that is, a healthy or positive change. From the bivariate analyses, variables with a p-value < 0.2 were retained for further analysis [42]. A final logistic regression was conducted to determine the strength of the associations between these variables and change in smoking. Results are presented as odds ratio (ORs) with 95% confidence interval (CI). P-values < 0.05 were considered statistically significant. Analyses were performed with IBM SPSS Statistics, version 27.

## Results

Among 335 respondents included in the analysis, 175 were from the outpatient OMT group, and 160 were from the inpatient group (Table 1). At T0, patients in the OMT group reported opioids as the most used substance group (78%). Among the inpatient group, the substances most used were stimulants (38%) and cannabis (28%). Patients in both groups reported polysubstance use 4 weeks prior to treatment, with a mean above 2 drugs for both groups. Both groups had a mean score on the lower side of the self-reported physical health scale. Patients did however report only modest levels of respiratory ailments. Asthma was reported by roughly 1 in 5 in the OMT group, and by about 1 in 8 among in the inpatient group. Sociodemographic variables were generally similar between the two groups. Around half had completed secondary education or higher. Only a minority reported being employed or enrolled in education. There was no significant difference between the groups’ HSCL-25 scores, but both groups had means above 1.0, suggesting levels of mental distress at T0 of clinical concern. The mean SDS scores were > 10 for both groups, indicating a high level of substance use severity. The major differences between the groups were that patients in OMT were roughly 10 years older than the inpatients and reported more stable living conditions 4 weeks prior to start of treatment (Table 1).

**Table 1:** Baseline demographics and other relevant variables of the patients that were reached at the 1-year follow-up grouped by OMT and other SUD inpatient treatment

			OMT (N = 175)		Inpatient (N = 160)		p-value^a^;		All patients (N = 335)	
***Sociodemographics***										
Age		M (SD)	39 (10)		29 (7)		**< 0.001** ^b^		34 (10)	
Male		n (%)	127 (73)		110 (69)		0.443		237 (71)	
2° education or higher		n (%)	83 (47)		65 (41)		0.230		148 (44)	
Employed/education		n (%)	25 (14)		21 (13)		0.728		46 (14)	
Stable living conditions		n (%)	147 (84)		111 (69)		**0.002**		258 (77)	
***Somatic health***										
Asthma		n (%)	31 (18)		20 (13)		0.249		51 (15)	
Respiratory ailments^c^		M (SD)	0.76 (1.16)		0.83 (1.16)		0.550		0.79 (1.16)	
Physical health^d^		M (SD)	1.58 (1.05)		1.65 (1.09)		0.527		1.61 (1.07)	
***Mental health and other variables***										
Mental distress^e^		M (SD)	1.26 (0.87)		1.22 (0.74)		0.604		1.24 (0.81)	
Severity of dependence^f^		M (SD)	10.36 (3.17)		10.08 (3.06)		0.536		10.23 (3.12)	
^a^ p-values are based on t-tests and chi-square tests ^b^ Equal variance not assumed ^c^ Item from somatic complaints questionnaire ^d^ Item from QOL10 ^e^ HSCL-25 ^f^ SDS										

**Table 2:** Smoking and tobacco use among OMT patients (N = 175) and other SUD treatment inpatients (N = 160) from treatment start to 1-year follow-up

Tobacco		OMT			Inpatient		
		T0	T1	p-value^a^;	TO	T1	p-value^a^;
Smoking	n (%)	165 (94)	153 (87)	< 0.001	149 (93)	110 (69)	< 0.001
Cigarettes	Mdn (IQR)	15 (10)	10 (10)	< 0.001	13 (13)	6 (13)	< 0.001
Smokeless tobacco	n (%)	47 (27)	59 (34)	0.074	66 (41)	83 (52)	0.023
Days/box	Mdn (IQR)	4 (5)	4 (5)	0.123	3 (4.75)	3 (3)	0.347

Notes: Cigarettes, daily cigarettes smoked; Days/box, days required to finish one box of smokeless tobacco

^a^ p-values based on McNemar’s test and Wilcoxon signed-rank test

In the full sample of 335 patients there was a significant (p < 0.001) decrease in smoking from T0 (94%) to T1 (79%). There was also a significant (p < 0.001) decrease in cigarette use, from a median of 15 cigarettes per day at T0 to a median of 10 cigarettes per day at T1. For smokeless tobacco, we found a significant (p = 0.003) increase in use from T0 (34%) to T1 (42.5%).

The within-group changes from T0 to T1 are shown in Table 2. We found a small albeit statistically significant (p < 0.001) decrease among the 175 OMT patients, as 12 (7%) patients reported smoking cessation, 10 (6%) continued as non-smokers, and 153 (87%) still reported smoking. Among 160 inpatients a significant (p < 0.001) decrease was also found, with 45 (28%) reporting smoking cessation, 5 (3%) remaining non-smokers, 6 (4%) starting to smoke, and 104 (65%) reporting that they were still smoking. Daily cigarettes smoked decreased significantly (p < 0.001) in both groups. A median reduction of 5 daily cigarettes was observed in patients in OMT, and among the inpatients there was a median reduction of 7 daily cigarettes.

Of the 57 subjects in the full sample who reported smoking cessation, 22 (39%) started using smokeless tobacco, 21 (37%) continued their smokeless tobacco habit, 8 (14%) quit both cigarettes and smokeless tobacco, and 6 (10%) did not use or commence using smokeless tobacco. Thus, a total of 14 (4%) former tobacco smokers reported total tobacco abstinence at follow-up. When investigating within-group smoking cessation and smokeless tobacco use, it was found that of the 12 OMT patients who reported smoking cessation at T1, 11 (92%) commenced or kept their existing smokeless tobacco habit, and 1 (0.6%) reported total tobacco abstinence. Among the 45 inpatients who reported smoking cessation, 32 (71%) either transitioned to or kept their existing smokeless tobacco habit at T1, and 13 (9%) reported total tobacco abstinence.

**Table 3:** Logistic regression of relevant covariates for smoking cessation.^a^; (N = 335)

Variables	Bivariate analysis OR (95% CI)	p-value^a^;	Multivariate analysis OR (95%CI)	p-value^b^;
Age	0.90 (0.87–0.94)	**< 0.001**	0.93 (0.89–0.97)	**0.002**
Sex, Female	1.52 (0.84–2.77)	0.169	1.64 (0.85–3.17)	0.141
Education level	0.71 (0.40–1.26)	0.235		
Employed or in school	0.84 (0.35–1.98)	0.684		
Treatment, inpatient	5.32 (2.69–10.49)	**< 0.001**	2.71 (1.25–5.86)	**0.011**
Cigarettes per day	1.0 (0.97–1.02)	0.928		
Respiratory ailments^d^	1.11 (0.93–1.41)	0.378		
Physical health^e^	1.21 (0.93–1.59)	0.156	1.31 (0.98–1.76)	0.065
Mental distress^f^	0.82 (0.57–1.19)	0.300		
Severity of dependence^g^	0.99 (0.91–1.09)	0.866		
^a^ The dependent variable was dichotomized smoking change ^b^ p-value obtained from bivariate logistic regression ^c^ p-value obtained from multivariable logistic regression; multivariable analysis included variables with p-values < 0.20 in bivariate analyses ^d^ Item from somatic complaints questionnaire ^e^ Item from QOL10 ^f^ HSCL-25 ^ g^ SDS				

A logistic regression was performed to assess factors associated with smoking cessation at follow-up. Treatment modality and age were the only two variables that were significantly associated with smoking cessation (Table 3). The odds of reporting smoking cessation were nearly 3-fold higher for inpatients than for OMT-patients in the adjusted model. Higher age was negatively associated with smoking cessation.

## Discussion

There was a very high prevalence of smoking among patients entering SUD treatment, and a low cessation rate 1 year after admission. Higher age and being in the OMT group (versus the inpatient group) were associated negatively with quitting smoking.

The high prevalence of smoking among patients entering treatment was observed during a period in which smoking had been on a decline for years in the Norwegian general population, to around 13% daily smokers in 2015 [13]. This is consistent with previous research that suggested smoking prevalence is still extremely high among patients with SUDs [43]. We interpret these findings to highlight the struggles with inherent challenges faced by individuals with OUD and other SUDs.

When we investigated change in smoking by treatment modality, we found that most patients in OMT were smokers both at the start of treatment and after about 1 year of treatment. This is consistent with findings from a relatively recent systematic review on smoking prevalence in addiction treatment [8]. Among inpatients, the numbers were similar to those among OMT subjects at the start of treatment, but inpatient smoking cessation levels were higher at the 1-year follow-up. Despite the relatively low quit rate, the reported number of daily cigarettes was reduced significantly in both groups, similar to findings from previous research [44]. Although the number of patients reporting smoking cessation in OMT was relatively low, our findings suggest that it is possible for individuals in OMT to quit smoking.

When we investigated factors associated with smoking cessation, treatment modality and age were the two variables contributing significantly in the regression model. The treatment modality association may be partly explained by a difference in time of exposure to active psychosocial treatment between the out-patient OMT and inpatient SUD treatment of several months’ duration. OMT is often considered life-long [31] and may be referred to as a “long-term low-intensity treatment”. In the Norwegian OMT model, somatic health follow-up including smoking cessation counseling has often been organized as the responsibility of the patient’s general practitioner, with overall responsibility for OMT residing with the specialist healthcare service [31]. Inpatient treatment or residential treatment, in comparison, can be characterized as “shorter-term and higher-intensity treatment”. Inpatients may spend 6–9 months in an environment where goals revolve around lifestyle change as well as abstinence from substances. It may involve several social activities that increase the possibility of patients influencing each other in the recovery process. As such, the social environment in a residential treatment facility will perhaps have a stronger impact on tobacco habits than will the environment in OMT treatment, where patients relate less to other patients. We note that differences between the patients in the two treatment modalities—namely, differences in age, stability of living conditions, and primary underlying SUDs—may also have contributed to the observed differences in smoking cessation between modalities.

The age association, although modest, indicated that smoking cessation was less likely to occur as patients aged. This is concordant with findings in general population samples that have indicated that as individuals age, their intention to quit smoking decreases and their nicotine dependence increases [45]. Along similar lines, positive associations between age and being a hardcore smoker (i.e., less inclined to quit) have also been reported [46, 47]. In other words, having a long history of tobacco use and being a hardcore smoker may be confounders for age. The literature on the relationship between age and smoking in SUD populations is sparse [48]. Nevertheless, with the increasing age of the OMT population it appears that treatment providers need to be mindful of age when considering smoking counseling and treatment, independent of where in the treatment trajectory an individual resides.

An interesting observation made relates to smokeless tobacco use. We found that most patients that quit smoking kept their existing smokeless tobacco habit or started using smokeless tobacco during the follow-up period. Smokeless tobacco has previously been described as a means of quitting for individuals already smoking in the general population [49, 50]. Tobacco abstinence would usually be the recommended outcome. However, given the difference in risk for developing health problems between smoking and smokeless tobacco, the transition from smoking to smokeless tobacco may be considered an improvement from a harm reduction perspective [51, 52].

It has previously been noted that there appears to be an unmet need for smoking cessation interventions in the addiction services [53, 54]. Our findings support this notion in the time frame of our data collection (2012–2016), especially within OMT. In 2018, after the start of the present study, standardized clinical pathways for mental health and SUD were published by the Norwegian directorate of health [55]. Improved somatic health follow-up was noted as a specific aim [55]. The aim of these standardized clinical pathways is to ensure that patients receive the same quality level of treatment and care, independent of, for example, geographical location. Even though a large part of the clinical pathways relates to quality outcomes, patient logistics, and structure, they also include recommendations and criteria of direct relevance to clinical practice and treatment outcomes. Included within the structure of the standardized clinical pathway are specific recommendations for somatic health check-ups [56], as well as recommendations on how to address smoking and smoking cessation. At the present time, not much is known about to the degree to which these clinical pathway recommendations have affected clinical practice with regard to somatic health, or if they have affected smoking cessation among patients currently enrolled in treatment. It will be of interest to researchers, decision makers, and clinicians to monitor smoking rates and cessation rates in SUD populations in the years to come, concurrent with the further implementation of the national guidelines and standardized clinical pathways.

### Strengths and limitations

A strength of this study is its prospective design employing data from a relatively large clinical cohort. The data were collected through interviews, with a low prevalence of missing data. The first interviews were conducted in a treatment setting, and the follow-up interviews were conducted by independent researchers external to the treatment process. The use of external researchers would lessen the risk of patient response bias, but likely not eliminate it completely. Some limitations should be considered when interpreting the results. With self-reported measurements there is always a chance of social desirability and recall biases. Results should thus be interpreted with caution, as self-reported smoking status was not independently verified. Furthermore, we do not have data on whether formal smoking cessation interventions and smoking cessation aids were provided as part of treatment, nor whether smoking cessation benefits were communicated. Future studies should include questions concerning smoking cessation motivation among patients in order to increase our understanding of the mechanisms underlying the cessation outcomes. We note also that, due to the inherent differences between the OMT and inpatient groups, the findings should be used to put the results from OMT into context and not as a direct comparison.

## Conclusion

As illustrated by the high smoking prevalence and relatively low cessation levels in our sample, an increased focus on smoking cessation among patients currently in OMT is warranted. Harm reduction–oriented smoking interventions (e.g., smokeless tobacco) may also be of relevance and interest for future research.

## Electronic supplementary material

Below is the link to the electronic supplementary material.

Supplementary Material 1. NorComt structured interview (excerpts)

## Data Availability

The dataset used during the current study is available from the corresponding author on reasonable request.

## Declarations

### Clinical trial registration

Clinicaltrials.gov no. NCT05182918. Registered 10/01/2022, i.e., the study was retrospectively registered

## List of abbreviations

EuropASI: Addiction Severity Index adapted for European use.
HSCL-25: Hopkins Symptom Checklist 25.
IQR: Interquartile range.
Mdn: Median.
NorComt: Norwegian Cohort of Patients in Opioid Maintenance Treatment and Other Drug Treatment.
OMT: Opioid maintenance treatment.
OUD: Opioid use disorder.
QOL10: Ten-item general quality of life instrument.
SD: Standard deviation.
SDS: Severity of Dependence Scale.
SUD: Substance use disorder.
