# Supplementary material for: High smoking and low cessation rates among patients in treatment for opioid and other substance use disorders
Source: BMC Psychiatry. 2022 Oct 19;22:649. doi: 10.1186/s12888-022-04283-6 (PMC9583489; doi:10.1186/s12888-022-04283-6)
Supplement: Supplementary file 1 — Supplementary Material 1. NorComt structured interview (excerpts) [file 12888_2022_4283_MOESM1_ESM.pdf]

NorComt structured interview (excerpts)[1]

Treatment	
	1 = Opioid maintenance treatment
	2 = Inpatient SUD treatment

**Sociodemographics**			
Age:			
Sex:		1 = Male	2 = Female
**Highest level of education completed**			
1 = Not finished primary			
2 = Primary			
3 = Secondary			
4 = Post-secondary vocational school			
5 = Tree years of higher education (college/university)			
6 = More than three years of higher education (college/university)			
9 = Unknown			
**Employment status**			
1 = Not employed and not enrolled in education			
2 = Full time employment			
3 = Part time employment			
4 = Enrolled in education			
5 = Part time employment and enrolled in education			
**Stable living conditions past 4 weeks**			
1 = Yes			
2 = No			
9 = Unknown			

[1] Excerpts from the NorComt structured interview used at baseline (T0), including the variables presented in this study. A similar structured interview was used at follow-up (T1). These excerpts has been translated from Norwegian to English.

**Substance use past 6 months (before controlled environment/treatment):**		
	**Type:**	**Use past 4 weeks:**
**Most used substance**		
**2nd most used substance**		
**3rd most substance**		
**4th most used substance**		
0 = Not used		1 = Not used
1 = Alcohol		2 = Less than once a week
2 = Cannabis		3 = Weekly
3 = Heroin/Opium		4 = 2-4 days per week
4 = Methadone, buprenorphine, other opiates/opioids prescribed in an OMT-program		5 = 5-6 days per week
5 = Methadone, buprenorphine, other opiates/opioids prescribed outside an OMT-program		6 = Daily
6 = Methadone, buprenorphine, other opiates/opioids obtained without prescription.		9 = Unknown
7 = Benzodiazepines, prescribed		
8 = Benzodiazepines, not prescribed		
9 = Other addictive medications		
10 = Amphetamines		
11 = Cocaine		
12 = Crack		
13 = Other stimulants		
14 = LSD or similar		
15 = Ecstasy		
16 = Inhalants/Solvents		
17 = Technical ethanol or equivalent		
18 = Other		
99 = Unknown		

**Somatic Health 1. How much have you been bothered by: (past 6 months, before treatment)**					
	0	1	2	3	4
	Not at all	A little	Moderately	A lot	Very much
Respiratory ailments					

**Somatic Health 2.**			
Are you currently suffering from asthma?	Yes	No	Unknown

**Somatic Health 3.**						
	n/a	1	2	3	4	5
How did you consider your physical health before entering treatment		Very poor	Poor	Neither good nor bad	Good	Very good

SCL-25. How much have you been bothered by: (for the past week week)<br>(Same questions as SCL-90)					
	0<br>Not At All	1<br>A Little Bit	2<br>Moderately	3<br>Quite A bit	4<br>Extremely
1. Headaches	0	1	2	3	4
2. Trembling	0	1	2	3	4
3. Faintness	0	1	2	3	4
4. Nervousness	0	1	2	3	4
5. Being scared for no reason	0	1	2	3	4
6. Feeling fearful	0	1	2	3	4
7. Heart racing	0	1	2	3	4
8. Feeling tense	0	1	2	3	4
9. Feeling panic	0	1	2	3	4
10. Feeling restless	0	1	2	3	4
11. Feeling low in energy	0	1	2	3	4
12. Blaming oneself	0	1	2	3	4
13. Crying easily	0	1	2	3	4
14. Thinking of ending one's life	0	1	2	3	4
15. Poor appetite	0	1	2	3	4
16. Sleep disturbance	0	1	2	3	4
17. Feeling hopeless	0	1	2	3	4
18. Feeling blue	0	1	2	3	4
19. Feeling lonely	0	1	2	3	4
20. Losing sexual interest	0	1	2	3	4
21. Feeling trapped	0	1	2	3	4
22. Worrying too much	0	1	2	3	4
23. Feeling no interest	0	1	2	3	4
24. Feeling that everything is an effort	0	1	2	3	4
25. Worthless feeling	0	1	2	3	4

**Severity of Dependence Scale (SDS)**				
Before you entered treatment:				
	Never	Sometimes	Often	Always
Did you think your use of substances was out of control?	0	1	2	3
Did the prospect of missing a fix (or dose) or not chasing make you anxious or worried	0	1	2	3
Did you worry about your use of substances?	0	1	2	3
Did you wish you could stop?	0	1	2	3
	Not difficult	Quite difficult	Very difficult	Impossible
How difficult would you find it to stop or go without substances? (Excluding OMT-medication)	0	1	2	3

**Tobacco use past 6 months before treatment**				
**Did you smoke tobacco?**			**Did you use snus (smokeless tobacco)?**	
	1 = Yes			1 = Yes
	2 = No			2 = No
If yes, how many cigarettes per day?			If yes, how many days per box?	
Answer:			Answer:	
